# Impact of myelodysplasia-related gene mutations and residual mutations at remission in venetoclax/azacitidine for AML

**DOI:** 10.1038/s41375-025-02625-3

**Published:** 2025-04-21

**Authors:** Yoshikazu Ikoma, Nobuhiko Nakamura, Yuto Kaneda, Hiroyuki Takamori, Tomokazu Seki, Nobuhiro Hiramoto, Junichi Kitagawa, Junya Kanda, Kei Fujita, Tetsuji Morishita, Yotaro Ochi, Shigeru Chiba, Nana Sasaki, Michiko Ichii, Kazunori Imada, Mizuki Watanabe, Masakatsu Hishizawa, Yasuko Miyahara, Yoshitomo Maesako, Yasuhiro Tanaka, Satoko Oka, Masaaki Tsuji, Satoshi Yoshihara, Kinuko Mitani, Yasunori Ueda, Toshiyuki Kitano, Mitsumasa Watanabe, Nobuo Sezaki, Tadakazu Kondo, Senji Kasahara, Akifumi Takaori-Kondo, Nobuhiro Kanemura, Seishi Ogawa, Yasuhito Nannya

**Affiliations:** 1 Department of Hematology and Infectious Disease, Gifu University Hospital, Gifu, Japan; 2 Division of Hematopoietic Disease Control, The Institute of Medical Science, The University of Tokyo, Tokyo, Japan; 3 Department of Hematology, Kobe City Medical Center General Hospital, Hyogo, Japan; 4 Department of Hematology, Gifu Municipal Hospital, Gifu, Japan; 5 Department of Hematology, Graduate School of Medicine, Kyoto University, Kyoto, Japan; 6 Department of Internal Medicine, Matsunami General Hospital, Gifu, Japan; 7 Healthcare Economics and Quality Management, Kyoto University, Kyoto, Japan; 8 Department of Pathology and Tumor Biology, Kyoto University, Kyoto, Japan; 9 Department of Hematology, Faculty of Medicine, University of Tsukuba, Tsukuba, Japan; 10 Laboratory of Stem Cell Therapy, Institute of Medicine, University of Tsukuba, Ibaraki, Japan; 11 Division of Hematology, Mito Saiseikai General Hospital, Ibaraki, Japan; 12 Department of Hematology, Japanese Red Cross Kyoto Daini Hospital, Kyoto, Japan; 13 Department of Hematology and Oncology, Osaka University Graduate School of Medicine, Osaka, Japan; 14 Department of Hematology, Japanese Red Cross Osaka Hospital, Osaka, Japan; 15 Department of Hematology, National Cancer Center Hospital, Tokyo, Japan; 16 Department of Hematology, Kyoto-Katsura Hospital, Kyoto, Japan; 17 Department of Hematology, Kyoto City Hospital, Kyoto, Japan; 18 Department of Hematology, Takatsuki Red Cross Hospital, Takatsuki, Japan; 19 Department of Hematology, Shinko Hospital, Kobe, Japan; 20 Department of Hematology, Japanese Red Cross Wakayama Medical Center, Wakayama, Japan; 21 Department of Hematology and Immunology, Japanese Red Cross Otsu Hospital, Otsu, Japan; 22 Department of Hematology, Hyogo Medical University Hospital, Hyogo, Japan; 23 Department of Hematology and Oncology, Dokkyo Medical University, Tochigi, Japan; 24 Department of Hematology/Oncology, Kurashiki Central Hospital, Kurashiki, Japan; 25 Department of Hematology, Medical Research Institute Kitano Hospital, Osaka, Japan; 26 Department of Hematology, Hyogo Prefectural Amagasaki General Medical Center, Hyogo, Japan; 27 Department of Hematology, Chugoku Central Hospital, Hiroshima, Japan; 28 Laboratory of Pharmaceutical Health Care and Promotion, Gifu Pharmaceutical University, Gifu, Japan

**Keywords:** Acute myeloid leukaemia, Cancer genetics

## Abstract

Venetoclax plus azacitidine (VEN + AZA) is widely used in acute myeloid leukemia (AML). This study explored the role of static and dynamic profiles of mutational clonal burden to predict outcomes by analyzing marrow samples from 228 VEN + AZA treated AML cases at “Pre-treatment” (*n* = 228), “Best-response” (*n* = 105), and “Relapse” (*n* = 27) phases using targeted-capture sequencing. In a multivariate model, older age, prior AZA, *TP53* mutation with variant allele frequency ≥0.10, and RAS-pathway mutations predicted shorter overall survival (OS), while *BCORL1* mutation predicted longer OS. Notably, myelodysplasia-related gene mutations, which constitute adverse factors in ELN 2022, predicted favorable survival. Achieving composite complete remission (CRc) significantly predicted longer OS (*P* < 0.001) but showed residual mutations in 76.2% of the cases. Among CRc cases, relapse-free survival was stratified by molecular clearance of mutations other than *DNMT3A*, *ASXL1*, and *TET2* (*P* = 0.04). In addition, 37% of relapsed cases showed a change of major clones, with 40% having potential targets of molecular-targeting treatment. This study revealed the novel prognostic role of myelodysplasia-related gene mutations and established the importance of molecular response assessment in CRc phase.

Venetoclax combined with azacitidine (VEN + AZA) has become the standard treatment for acute myeloid leukemia (AML) patients unsuitable for intensive chemotherapy [1–5]. However, in practice, VEN + AZA is also applied to relapsed or refractory (R/R) AML as salvage treatment [6–9]. VEN + AZA treatment outcome is inadequately predicted by The European LeukemiaNet (ELN) risk classification, which assign the cases with myelodysplasia-related gene (MR-gene) mutations as high-risk [10–13]. Recently, a VEN + AZA-specific prognostic model, incorporating *TP53*, RAS-pathway mutations, and *FLT3*-ITD was proposed [11], but focused on front-line setting alone. More importantly, the role of measurement of post-treatment genetic burden has not been studied. To address these issues, we retrospectively studied 228 AML patients who received VEN + AZA treatment between March 2021 and July 2023. Genomic DNA was extracted from bone marrow (BM) samples at three defined timepoints: “Pre-treatment”, “Best-response”, and “Relapse” (Supplementary Methods) and genetic profile including single-nucleotide variations (SNVs), copy number alterations (CNAs), and structural variations was obtained by targeted-capture sequencing (Supplementary Table 1, Supplementary Methods). This study was conducted in accordance with the Declaration of Helsinki and was approved by the Institutional Ethical Committees at Kyoto University (G-608, G-697) and all the participating institutes. All patients provided written informed consent.

This cohort (*n* = 228) included 118 (51.8%) cases of AML with myelodysplasia-related changes (AML-MRC) [14], 111 (48.7%) untreated cases, and other data are shown in Supplementary Tables 2, 3, 4. We collected 360 specimens with 228, 105, and 27 specimens being collected from Pre-treatment, Best-response, and Relapse phases, respectively (Supplementary Fig. 1A, B). Targeted-capture sequencing identified a total of 2293 genetic alterations (1043 SNVs and short indels, 1195 CNAs, and 55 structural variations, Supplementary Fig. 1C–E Supplementary Tables 5, 6, 7). *TP53* mutation was categorized into *TP53*^high^ and *TP53*^low^ based on the largest *TP53*-mutation size with a cutoff of 0.10 (Supplementary Fig. 2) [10].

Among the pre-treatment samples, -7/del(7q) were the most frequent events (23.2%), followed by *TP53*^high^ (21.9%), -5/del(5q) (21.5%), *RUNX1* (18.9%) and *TET2* (17.1%) (Fig. 1A). ELN 2022 risks were favorable (*n* = 29, 14.7%), intermediate (*n* = 29, 14.7%), and adverse (*n* = 139, 70.6%). 38.1% of the adverse-risk group were classified based on the involvement of MR-gene mutations (*ASXL1/BCOR/EZH2/RUNX1/SF3B1/SRSF2/STAG2/U2AF1/ZRSR2*) alone. Recently-proposed VEN + AZA specific risk predictions for upfront cases (VIALE-A risks thereafter) [11] were also assessed (Fig. 1B).

**Fig. 1:**
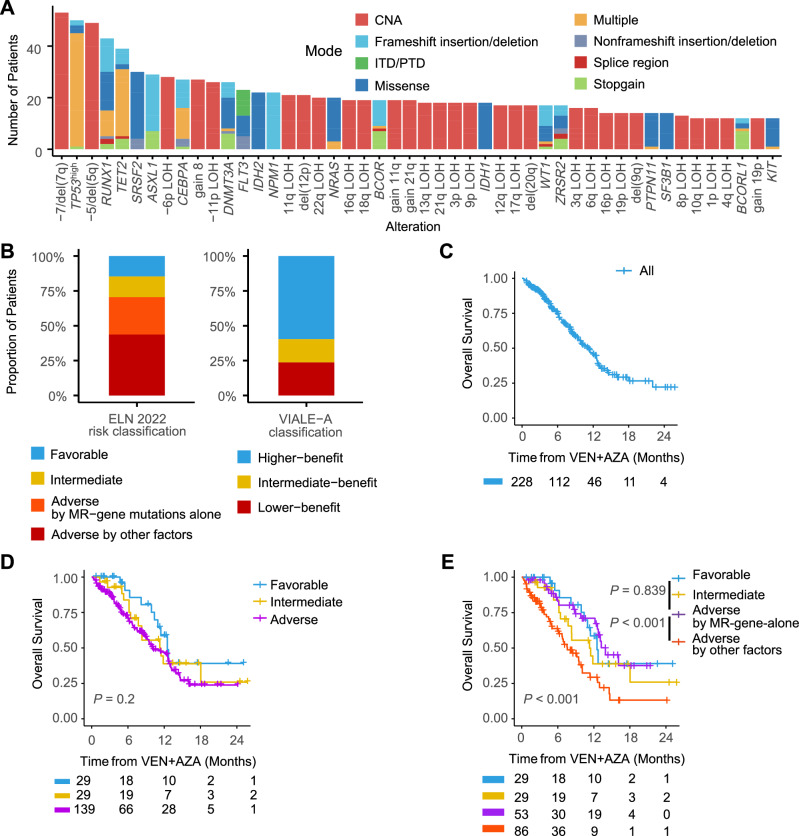
Clinical, genomic features, risk classification and OS analysis in AML patients treated with VEN + AZA. **A** Frequency of genetic alterations in pre-treatment samples. Bar plots show the frequency of genetic alterations in more than 5% of cases (*n* = 228). **B** ELN 2022 risk classification (left) and VIALE-A risk classification (right) of pre-treatment samples. Stacked bar plot showing the proportion of patients across different risk categories per the ELN 2022 classification (*n* = 197). The adverse risk group is divided into subcategories based on MR-gene mutations alone and other adverse factors. **C** Kaplan–Meier curve showing overall survival (OS) for all AML patients included in the study (*n* = 228) undergoing VEN + AZA treatment. **D** Kaplan–Meier curves for OS by ELN 2022 classification. OS curves stratified by ELN 2022 risk categories (*n* = 197). Groups include favorable, intermediate, and adverse risk, with adverse risk further detailed in (**E**). **E** Kaplan–Meier curves for OS according to ELN 2022 adverse subgroups. The adverse risk group is divided into two subcategories: patients classified as adverse due to the presence of myelodysplasia-related gene (MR-gene) mutations alone (MR-gene-alone) versus those with other adverse risk factors. CNA, copy number alterations; ITD/PTD, internal tandem duplication/partial tandem duplication; MR-gene, myelodysplasia-related gene (including mutations of *ASXL1, BCOR, EZH2, RUNX1, SF3B1, SRSF2, STAG2, U2AF1, ZRSR2*); MR-gene-alone, patients classified as adverse-risk due to MR-gene mutations alone.

CR, partial hematologic recovery (CRh), and CR with incomplete hematologic recovery (CRi) were obtained in 38.7%, 7.8%, and 16.6% of the cases with response data and referred to as CRc [1]. The number of VEN + AZA cycles and time to response are detailed in Supplementary Table 8. A multivariate analysis identified previous AZA treatment (OR: 0.28, 95% CI: 0.11–0.67, *P* = 0.005) and *TP53*^high^ (OR: 0.29, 95% CI: 0.10–0.78, *P* = 0.015) as adverse factors, and *ASXL1* (OR: 3.67, 95% CI: 1.07–17.46, *P* = 0.06) as a favorable factor for achieving CRc (Supplementary Fig. 4, Supplementary Table 9).

Median OS, progression-free survival (PFS), and relapse-free survival (RFS) for responders (*n* = 136) were 14.4 months, 10.8 months, and 14.8 months, respectively (Fig. 1C, Supplementary Fig. 5A, B). OS after VEN + AZA treatment was not successfully stratified with ELN 2022 risk classification, especially for newly diagnosed cases (Fig. 1D, Supplementary Fig. 3A, B). VIALE-A classification also failed to stratify intermediate- and lower-benefit groups (*P* = 0.858, log-rank test, Supplementary Fig. 3C), probably due to higher AML-MRC cases in this cohort. Notably, those assigned to the ELN 2022 adverse risk group based on the presence of MR-gene mutations alone (MR-gene-alone) had significantly longer OS compared to those who were assigned to the adverse risk group for reasons other than MR-gene mutations (*P* < 0.001, log-rank test, Fig. 1E). This effect was more remarkable in the R/R cases (Supplementary Fig. 3D, E) and has been supported by a previous report [11].

Thus, we further evaluated prognostic factors associated with OS after VEN + AZA treatment in which we included “MR-gene-alone” as a candidate covariate based on the observation above. The R/R cohort showed comparable OS to ND cases probably due to younger age and less AML-MRC in the R/R cohort (Supplementary Fig. 5C, Supplementary Table 3). The final model constructed by a multivariate Cox regression analysis included *BCORL1* (HR: 0.31, 95% CI: 0.11–0.88, *P* = 0.027), MR-gene-alone (HR: 0.69, 95% CI: 0.41–1.16, *P* = 0.159) as favorable factors, and high age (≥70) (HR: 1.63, 95% CI: 1.01–2.63, *P* = 0.044), previous AZA treatment (HR: 1.57, 95% CI: 0.98–2.53, *P* = 0.062), RAS-pathway genes (HR: 2.69, 95% CI: 1.64–4.39, *P* < 0.001), and *TP53*^high^ (HR: 2.14, 95% CI: 1.31–3.48, *P* = 0.002) as unfavorable prognostic factors (Supplementary Fig. 6, Supplementary Table 10).

CR and CRh/CRi groups showed similar OS (*P* = 0.739, log-rank test) and this trend was also observed for RFS and progression-free survival (Fig. 2A, B, Supplementary Fig. 7A). Univariate analysis showed that achieving CRc had much greater impact on OS compared to other pretreatment factors (HR: 0.23, 95% CI: 0.15–0.35, *P* < 0.001, Supplementary Fig. 7B). These results, combined with the fact that as many as 63.1% of this cohort achieved CRc, strongly support the need to stratify the CRc group. We had 89 cases who achieved CRc and had paired samples for both Pre-treatment and Best-response phases. 472 mutational events from 119 genes or CNAs were observed in pretreatment samples and 43 (%) disappeared in the CRc phase (Fig. 2C, Supplementary Fig. 7C). Persistent rates were much affected by mutational events (Fig. 2D). Notably, this cohort included 13 cases with *TP53* mutations in the Pre-treatment samples and 53.8% of them turned *TP53*-mutation negative in the CRc phase. However, molecular elimination of *TP53*-mutated clones did not result in prolonged OS (*P* = 0.39, log-rank test) and RFS (*P* = 0.97, log-rank test) (Supplementary Fig. 7D, E).

**Fig. 2:**
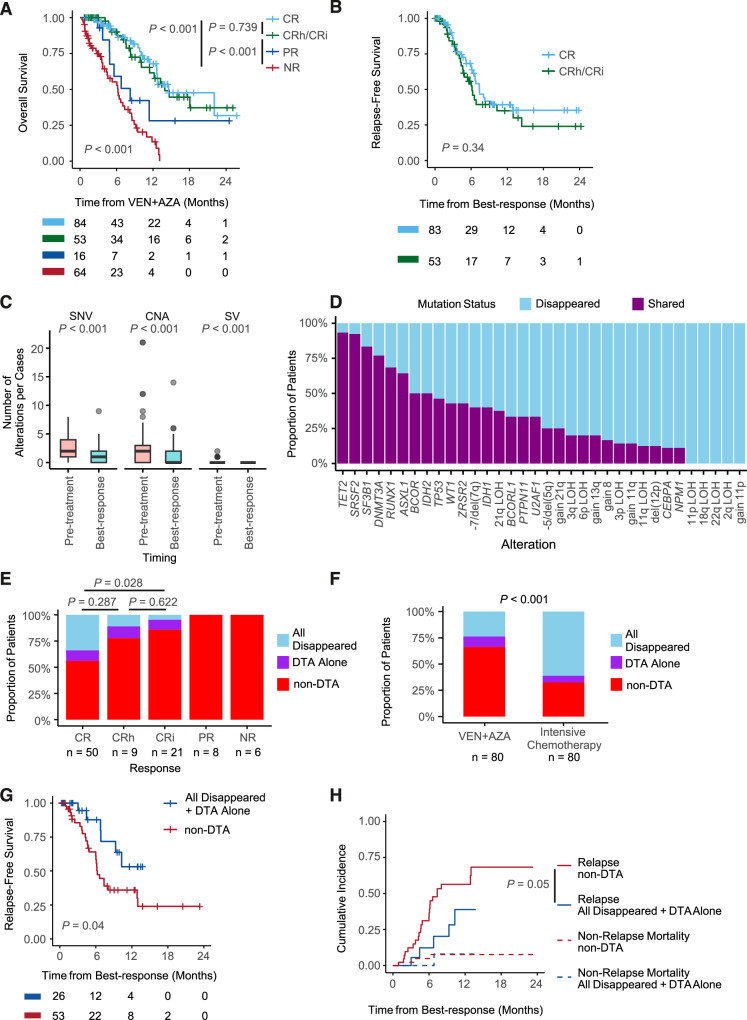
Mutational profile in CRc status and its effect on survival. Kaplan–Meier curves showing OS (**A**) or RFS (**B**) stratified by hematological response to VEN + AZA treatment. *P* values are calculated by log-rank test. **C** Box plots showing the number of genetic alterations per case (left: SNV, middle: CNA, right: SV) for the 89 cases that achieved CRc and had both Pre-treatment and Best-response samples analyzed. *P* values are calculated with paired-t test. **D** Bar charts showing the ratio of genetic alterations that were shared between Pre-treatment and Best-response samples. **E** Stacked bar charts showing the frequency of the cases having non-DTA mutations (red), DTA-mutations alone (purple), or no mutations (blue). The x-axis shows the hematological responses. The 121 patients who had any mutations in either Pre-treatment or Best-response were included in the analysis. *P* values were calculated using Fisher’s exact test to compare the ratio of cases with non-DTA mutations between response categories. **F** Stacked bar charts showing the difference of residual mutation status in CRc status for 80 cases who received VEN + AZA and 80 cases who received intensive chemotherapy. *P* values were calculated using Fisher’s exact test to compare the ratio of cases with non-DTA-mutations between treatment groups. **G** Kaplan–Meier curves showing RFS stratified by the residual mutations (red: residual non-DTA-mutations, blue: no residual mutations or DTA-mutations alone) in CRc status. *P*-values are calculated by log-rank test. **H** Cumulative incidence curves showing relapse (straight lines) or non-relapse mortality (dotted lines) for the same cohort as (**G**). OS overall survival, RFS relapse-free survival, SNV single nucleotide variants, CNA copy number alterations, SV structural variants, CRc composite complete remission, DTA, *DNMT3A*, *TET2*, and *ASXL1*.

We focused on the residual mutations in the CRc phase for the 80 cases who had at least one mutation in either Pre-treatment or Best-response phases (Supplementary Fig. 8A). Only 19 (23.8%) had no residual mutations and 8 (10%) had DTA-mutations alone. The remaining 53 (66.2%) had non-DTA mutations even in CRc response. CR cases had a significantly lower frequency of having non-DTA residual mutations compared to CRi (*P* = 0.028, Fig. 2E), suggesting that residual clones interfere with adequate blood recovery. The residual mutations were higher in VEN + AZA-treated cases when compared with another 80 cases who achieved CRc after intensive chemotherapy (idarubicin/daunorubicin with cytarabine) (Fig. 2F and Supplementary Fig. 8B, Supplementary Table 11). This difference suggests that VEN + AZA exerts its antineoplastic effects through a different mechanism than intensive chemotherapy.

The cases with residual DTA-clone alone have no impact on OS and RFS compared to those with complete molecular clearance (*P* = 0.63 for OS, *P* = 0.78 for RFS, log-rank test, Supplementary Fig. 9A, B). Therefore, we combined the cases with complete molecular clearance and residual DTA-clone alone into one group. This group showed significantly longer RFS compared to those with residual mutations in non-DTA genes (*P* = 0.04, log-rank test, Fig. 2G, H), although there was no significant difference in OS (Supplementary Fig. 9C). The lower relapse rate in the no or DTA-alone residual mutations group (*P* = 0.05, Fine & Gray test) explained the RFS benefit.

27 CRc cases had paired samples for both Pre-treatment and Relapse phases (Supplementary Fig. 10A). Increase of mutations in *TP53* (6 in Pre-treatment and 9 at Relapse) was the most striking feature in relapse (Supplementary Fig. 10B), which is consistent with a previous report [15]. Not surprisingly, *TET2*, *SRSF2*, *SF3B1*, and *DNMT3A* mutations, which were persistent in CRc status, also constituted leukemic clones at relapse. Clonal shifts between Pre-treatment and Relapse phases were categorized as either “clonal persistence” or “clonal change” patterns (Supplementary Methods). Clonal persistence, where the tumor clone at relapse was identical to the clone at baseline, was observed in 17 (63%) cases and*TP53*, *TET2*, *IDH1*, *RUNX1*, *and SF3B1* mutations constituted major clones (Supplementary Fig. 11A). Clonal changes, defined by replacement of major clones, were observed in 10 cases. In these cases, *TP53, CEBPA,* and *FLT3* mutations comprised the most frequent emerging clones (Supplementary Fig. 11B), and this was also compatible with a previous report [15]. The clonal evolution pattern did not affect OS nor RFS (OS: *P* = 0.79, RFS: *P* = 0.52, log-rank test, Supplementary Fig. 11C, D).

In conclusion, we have demonstrated that MR-gene mutations predict a favorable response to VEN + AZA. Furthermore, this study showed that 76.2% of CRc cases have a residual mutational burden, which is useful for further stratification of long-term outcomes of CRc cases.

## Supplementary information

Supplementary Information

Supplementary Tables

## Data Availability

Data collected for this study will be made available with publication to anyone who wishes to access the data for any purpose. This will include the protocol with all amendments, informed consent forms, complete de-identified patient data sets, and analytic or statistical code.
