# Supplementary material for: Impact of myelodysplasia-related gene mutations and residual mutations at remission in venetoclax/azacitidine for AML
Source: Leukemia. 2025 Apr 21;39(6):1362–7. doi: 10.1038/s41375-025-02625-3 (PMC12133561; doi:10.1038/s41375-025-02625-3)

Supplementary Information

Supplementary Methods

Patient eligibility and data collection

This retrospective study enrolled subjects from the hematological malignancy biobank at Kyoto University, collected from collaborative institutes in Japan. We identified acute myeloid leukemia (AML) cases treated with venetoclax plus azacitidine (VEN+AZA) from March 2021 to July 2023. Inclusion criteria of the patients were: (1) age 16 years or older, (2) AML diagnosis according to the WHO 2017 classification [1], and (3) fewer than five prior lines of treatment. In this context, one line of treatment was defined as follows: a) Induction therapy with anthracycline plus cytarabine followed by consolidation therapy (either with anthracycline plus cytarabine or high-dose cytarabine) was considered as one line of treatment. b) Hematopoietic stem cell transplantation was counted as one line of treatment. Other therapies were counted individually as separate lines of treatment. We excluded patients from the analysis if they were treated with VEN+AZA for palliative purposes or had a prior history of hematopoietic stem cell transplantation. Detailed patient characteristics, laboratory data, and treatment data and clinical outcomes were collected using a questionnaire from collaborative institutions. For comparison of residual mutations in composite complete remission (CRc) status, we also included 80 AML cases who achieved CRc after intensive chemotherapy.

Baseline parameter

Baseline parameters included patient age at VEN+AZA administration, sex, disease subtype according to WHO 2017 classification [1], karyotype, pre-existing hematologic disorders, disease status, and prior treatment history. For subsequent analyses, these parameters were dichotomized as follows: age into higher and lower groups by the median age of 70 years in the entire cohort, karyotype into adverse or non-adverse based on the European LeukemiaNet (ELN) 2022 risk classification [2], disease subtypes into AML with

myelodysplasia-related changes or others and AML with monocytic differentiation (FAB M4/M5) or others, disease status at VEN+AZA initiation into treatment-naive or previously treated and primary induction failure or others, and prior treatment history based on previous exposure to VEN, AZA, or FLT3 inhibitors.

Targeted sequencing

Sampling of tumor cells

Genomic DNA was extracted from bone marrow samples collected at three defined timepoints during the course of treatment. "Pre-treatment" was defined as the period prior to the start of VEN+AZA. This included newly diagnosed patients as well as previously treated patients. "Best-response" was defined as the time of the first bone marrow examination at which patients achieved their best response (either CRc or partial remission). If patients never achieved CRc or partial remission during the observation period, "Best-response" was defined as the time of optimal response while maintaining treatment feasibility. "Relapse" sample was defined as the first marrow examination documenting relapse in patients who had achieved CRc after VEN+AZA treatment.

Panel information for targeted-capture sequencing

We applied targeted-capture sequencing using a panel designed for genetic study of myeloid malignancies at Kyoto University. 445 genes were included in this panel and were investigated in this study (**Supplementary Table 1)**. This panel also included 1,428 SNPs probes so that we can detect genome-wide copy number changes and allelic imbalances.

Quality of panel sequencing

The median depth of the targeted capture sequencing was 498× (range, 169−765×). The depth profile of all the samples is presented in the figure below.

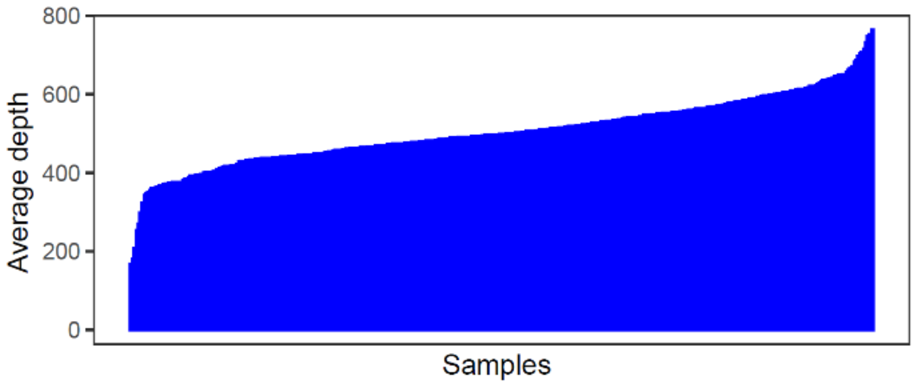

Sequencing method

All the samples were sequenced at Kyoto University. 50 ng or 200 ng of genomic DNA were enriched for target regions by liquid-phase hybridization using the SureSelect custom kit© (Agilent Technologies®), according to the manufacturer's protocol optimized for automated sample processing. The purified library was subjected to high-throughput sequencing analysis with NovaSeq 6000© (Illumina®) or DNBSEQ-G400© sequencer (MGI®) using 150 bp pair-end mode.

Mutation calling

Sequencing reads were aligned to the human genome reference (hg19) using Burrows-Wheeler Aligner, version 0.7.8 with default parameter settings. Mutation calling was performed through our established pipeline (genomon pipeline 2.6.3, https://github.com/Genomon-Project), as previously reported [3–6] using the following parameters.

Adopt variants fulfilling the following criteria:

(i) Mapping Quality score ≥ 20
(ii) Base Quality score ≥ 15
(iii) Number of total reads ≥ 100
(iv) Number of variant reads ≥ 4
(v) Variant allele frequency ≥ 0.02

(vi) Fisher's p-value for specific presentation compared with normal controls < 0.02

Following candidates were excluded:

(i) Synonymous and ambiguous (unknown) variants

(ii) Variants which were read only from one direction

(iii) Single nucleotide substitutions in which other mutations were called at the same position and their variant allele frequency was ≥ 0.1.

Mapping errors were removed by visual inspection on the Integrative Genomics Viewer (IGV) browser (http://software.broadinstitute.org/software/igv/). Structural variants were also called using genomon pipeline 2.6.3.

Curation of oncogenic variants

The significant variants that fulfill the quality filter noted above were further assessed for oncogenicity based on an in-house curation program. The curation policy was determined individually for each gene based on previous reports and databases after exclusion of variants registered in public SNPs databases (the 1000 genomes project as of 2014 Aug, ESP6500, Human Genome Variation Database) and call errors using EB call [7] and in-house blacklist of error calls. All the pathogenic calls are listed in **Supplementary Tables 5, 6, and 7**.

Definition of gene mutation groups

TP53-mutated cases were categorized into "$TP53^{high}$" and "$TP53^{low}$" groups based on variant allele frequency (VAF) with cutoff value of 0.10. *NRAS*, *KRAS*, *PTPN11*, *CBL*, and *NF1* mutations were grouped as "RAS-pathway genes". Myelodysplasia-related (MR) gene mutations include *ASXL1, BCOR, EZH2, RUNX1, SF3B1, SRSF2, STAG2, U2AF1, and ZRSR2* according to ELN 2022 risk classification system [2]. *DNMT3A*, *TET2*, and *ASXL1* mutations were collectively termed as "DTA-mutations", which are known to persist in remission and have age-related clonal hematopoiesis [8,9].

Copy number and allelic imbalance

We included 1,428 SNPs probes to allow for detection of copy number changes and allelic imbalances. This technique, called CNACS, is implemented in the program available at https://github.com/papaemmelab/toil_cnacs. Manual inspection of the result was conducted to discriminate call errors.

Total copy number (TCN) of 2.22 or larger was assumed CN-gains, and TCN <1.88 was assumed CN-loss. Copy number neutral LOH was called when B-allele frequency was <0.90 with TCN between 1.88 and 2.22. Arm level copy number alteration (CNA) were called when the total length of the affected region within the arm is > 1 M bp for 17p and >3 M bp for the other arms. For gains in chromosome 8, CNA events in long and short arms are counted together. Partial tandem duplication in *KMT2A* (*KMT2A*-PTD) was detected using CNA data. Specifically, *KMT2A*-PTD was called when copy number gain (TCN > 2.5) was detected within the region between intron 1 and intron 19 of *KMT2A* (NM_005933) gene.

Calculation of mutation clone size

Size of VAF of point mutations were adjusted accounting for copy number alterations or allelic imbalances and adjusted VAF values (adjVAF) were calculated, which represent the fraction of cells having relevant point mutations. The details of adjustment calculation are described before [10].

Definition of major clones (MC)

In the analysis of the clonal changes, *FLT3*-ITD and *KMT2A*-PTD were removed from the mutation list because of the difficulty in correctly estimating clone size for these mutations. The germline *DDX41* mutations were also removed. All the mutations were classified into MC or non-MC based on their VAF. If the difference between a clone's adjVAF and the maximum adjVAF in the same sample was less than 0.10, it was classified as a major clone; otherwise, it was categorized as a minor clone.

Statistical analysis

Definition of response and survival outcomes

Treatment response was determined according to modified International Working Group criteria [11]. CRc was defined as the sum of complete remission (CR), CR with partial hematologic recovery (CRh), and CR with incomplete hematologic recovery (CRi) [12]. Overall survival (OS) was defined as the time from start of VEN+AZA treatment to death from any cause. Relapse-free survival (RFS) was defined as the time from achieving CRc to AML relapse per ELN 2022 guidelines [2]. Progression-free survival (PFS) was defined as the time from the first response (including CRc and partial response) to disease progression or relapse. Survival time was estimated using the Kaplan-Meier method, with transplantation events treated as censored data in the analysis of OS, PFS, and RFS. Cox proportional hazards analysis was used for survival comparisons, although the log-rank test was additionally performed when noted in the literature. In cases where comparisons were made between three or more groups, pairwise analyses were conducted with Bonferroni correction applied to adjust for multiple comparisons.

Univariate analysis and parameter selection in multivariate analysis

Univariate analyses were conducted with Fisher's exact test for response analyses and Cox regression for survival analyses including OS, PFS, and RFS.

The variables with significant or sub-significant impact (p-values < 0.1) in univariate analyses were subjected to multivariate analyses. The variables that represent only a small portion of the cohort (< 10% for CNA factors and < 5% for the others) were also excluded from the multivariate analyses. The generalized variance inflation factor (gVIF) was calculated for covariates in the regression model to avoid multiple collinearities. If any variables had gVIF exceeding 2, the variable with the second-highest gVIF was removed and gVIF was recalculated, and this process was repeated until all remaining variables had a gVIF of 2 or less. Subsequently, Lasso regression was applied for variable selection, with the optimal regularization parameter (lambda) determined through cross-validation to prevent overfitting.

The lambda value yielding the lowest binomial deviance was selected to construct the most predictive model. For the final multivariate analyses, logistic regression models were applied to evaluate the impact of selected factors on treatment response, calculating odds ratios (OR) and 95% confidence intervals (CI). Cox regression models were applied for survival analyses, calculating hazard ratios (HR) and 95% confidence intervals (CI).

All statistical analyses were performed using R (version 4.3.1). In addition, R packages ggplot2_3.5.1, survminer_0.4.9, forestmodel_0.6.2, cmprsk_2.2-12, prodlim_2024.06.25, cowplot_1.1.3, and patchwork_1.2.0 were used for graphical presentation.

References

1. Swerdlow SH, Campo E, Harris NL, Jaffe ES, Pileri SA, Stein H, et al. (eds). WHO Classification of Tumours of Haematopoietic and Lymphoid Tissues. Revised 4th ed. IARC Publications, Lyon, 2017.
2. Döhner H, Wei AH, Appelbaum FR, Craddock C, DiNardo CD, Dombret H, et al. Diagnosis and management of AML in adults: 2022 recommendations from an international expert panel on behalf of the ELN. Blood. 2022;140:1345–77.
3. Yoshida K, Sanada M, Shiraishi Y, Nowak D, Nagata Y, Yamamoto R, et al. Frequent pathway mutations of splicing machinery in myelodysplasia. Nature. 2011;478:64–9.
4. Haferlach T, Nagata Y, Grossmann V, Okuno Y, Bacher U, Nagae G, et al. Landscape of genetic lesions in 944 patients with myelodysplastic syndromes. Leukemia. 2014;28:241–7.
5. Yoshizato T, Dumitriu B, Hosokawa K, Makishima H, Yoshida K, Townsley D, et al. Somatic mutations and clonal hematopoiesis in aplastic anemia. N Engl J Med. 2015;373:35–47.
6. Suzuki H, Aoki K, Chiba K, Sato Y, Shiozawa Y, Shiraishi Y, et al. Mutational landscape and clonal architecture in grade II and III gliomas. Nat Genet. 2015;47:458–68.
7. Shiraishi Y, Sato Y, Chiba K, Okuno Y, Nagata Y, Yoshida K, et al. An empirical Bayesian framework for somatic mutation detection from cancer genome sequencing

data. Nucleic Acids Res. 2013;41:e89.

8. Genovese G, Kähler AK, Handsaker RE, Lindberg J, Rose SA, Bakhoum SF, et al. Clonal hematopoiesis and blood-cancer risk inferred from blood DNA sequence. N Engl J Med. 2014;371:2477–87.

9. Jongen-Lavrencic M, Grob T, Hanekamp D, Kavelaars FG, Al Hinai A, Zeilemaker A, et al. Molecular minimal residual disease in acute myeloid leukemia. N Engl J Med. 2018;378:1189–99.

10. Yoshizato T, Nannya Y, Atsuta Y, Shiozawa Y, Iijima-Yamashita Y, Yoshida K, et al. Genetic abnormalities in myelodysplasia and secondary acute myeloid leukemia: impact on outcome of stem cell transplantation. Blood. 2017;129:2347–58.

11. Cheson BD, Bennett JM, Kopecky KJ, Büchner T, Willman CL, Estey EH, et al. Revised recommendations of the International Working Group for Diagnosis, Standardization of Response Criteria, Treatment Outcomes, and Reporting Standards for Therapeutic Trials in Acute Myeloid Leukemia. J Clin Oncol. 2003;21:4642–9.

12. Perl AE, Martinelli G, Cortes JE, Neubauer A, Berman E, Paolini S, et al. Gilteritinib or chemotherapy for relapsed or refractory FLT3-mutated AML. N Engl J Med. 2019;381:1728–40.

Legends for Supplementary Figures

Supplementary Fig. 1: Study flow and supplementary information of sequencing and detected alterations.

(A) Patient cohort and sample collection timeline. A flowchart summarizing the clinical background of the 228 AML patients included in the study at the start of VEN+AZA treatment. Sampling timelines are presented for Pre-treatment (n = 228), Best-response (n = 105; CRc: n = 89, Other: n = 16), and Relapse stages (n = 27).

(B) Bar plot showing the distribution of the number of genomic examinations per patient.

(C) Total number of genetic alterations across all time points.

(D) Box plots showing the total number of genetic alterations (sum of SNV, CNA, and SV) at Pre-treatment (n=228), Best-response (n=105), and Relapse (n=27) phases.

(E) Breakdown of Supplementary Fig. 1D into different genetic alterations.

AML, acute myeloid leukemia; PIF, primary induction failure; VEN+AZA, venetoclax plus azacitidine; OS, overall survival; CRc, composite complete remission; RFS, relapse-free survival; SNV, single nucleotide variants; CNA, copy number alterations; SV, structural variants.

Supplementary Fig. 2: Comparison of *TP53* mutation allelic status versus variant allele frequency (VAF) thresholds to be applied in outcome analyses for AML patients treated with VEN+AZA.

(A) *TP53* mutations were categorized based on allelic status as either multi-hit (n = 41) or monoallelic (n = 10), and by VAF into $TP53^{high}$ (VAF ≥ 0.1, n = 47) and $TP53^{low}$ (VAF < 0.1, n = 4) following Döhner et al., (Blood 2022, 140(12): 1345-).

(B) Response rates stratified by *TP53* VAF threshold. Stacked bar plots show the response rates to VEN+AZA treatment for patients stratified by *TP53* VAF. Categories include $TP53^{high}$ (VAF ≥ 0.1) and $TP53^{low}$ (VAF < 0.1). No significant difference was observed in CRc rates between $TP53^{high}$ and $TP53^{low}$ groups (*P* = 0.303, Fisher's exact test).

(C) Response rates stratified by *TP53* allelic status. Stacked bar plots showing the response rates to VEN+AZA treatment for patients categorized by *TP53* allelic status as multi-hit or monoallelic. There was a significant difference in CRc rates between these groups, with the monoallelic group showing higher response rates (P = 0.013, Fisher's exact test).

(D) OS stratified by *TP53* VAF threshold. Kaplan-Meier curves for OS in patients stratified by *TP53* VAF, comparing $TP53^{high}$ (VAF ≥ 0.1) and $TP53^{low}$ (VAF < 0.1) groups. Although not statistically significant (P = 0.075, log-rank test), patients in the $TP53^{low}$ group tend to have better overall survival.

(E) OS stratified by *TP53* allelic status. Kaplan-Meier curves showing OS for patients with *TP53* mutations, categorized by allelic status into multi-hit or monoallelic. No significant difference in OS was observed between the groups (P = 0.23, log-rank test).

Based on the considerations above, we assumed that *TP53* risk classification for AML is better achieved by VAF level, than allelic status. This is compatible with Tim Grob et al., (Blood 2022, 139(15): 2347-).

VAF, variant allele frequency; VEN+AZA, venetoclax plus azacitidine; OS, overall survival; CRc, composite complete remission; CR, complete remission; CRh, complete remission with partial hematological recovery; CRi, complete remission with incomplete hematological recovery; PR, partial remission; NR, no response.

Supplementary Fig. 3: Risk classification and overall survival (OS) analyses in AML patients treated with VEN+AZA.

(A) Kaplan-Meier curves for OS stratified by ELN 2022 classification in newly diagnosed (ND) patients (n = 94, subset of (B)). Risk groups include favorable, intermediate, and adverse risk.

(B) Kaplan-Meier curves for OS stratified by ELN 2022 classification in relapsed/refractory (RR) patients (n = 103, subset of (B)). Risk groups include favorable, intermediate, and adverse risk.

(C) Kaplan-Meier curves by newly proposed VIALE-A risk classification. OS curves are divided into higher-benefit, intermediate-benefit, and lower-benefit groups.

(D) Kaplan-Meier curves for OS in ELN 2022 adverse-risk patients with ND AML (subcategory of adverse risk group from (C)), stratified by MR-gene-alone mutations versus other adverse factors.

(E) Kaplan-Meier curves for OS in ELN 2022 adverse-risk patients with RR AML (subcategory of adverse risk group from (D)), stratified by MR-gene-alone mutations versus other adverse factors.

VEN+AZA, venetoclax plus azacitidine; OS, overall survival; MR-gene, myelodysplasia-related gene; MR-gene-alone, patients classified as adverse-risk due to MR-gene mutations alone; ND, newly diagnosed; RR, relapsed/refractory.

Supplementary Fig. 4: Analysis workflow and variable selection for identifying factors associated with CRc in AML patients treated with VEN+AZA.

(A) A schematic flowchart summarizing the four-step analytical approach used to identify independent predictors of CRc. Initially, univariate Fisher's exact tests were performed, and variables with $P < 0.1$ and sufficient frequency (≥10% for CNA, ≥5% for other alterations) were selected. Secondly, multiple collinearity was resolved based on generalized variance inflation factors (gVIF). Thirdly, Lasso regression was applied. Finally, multivariate logistic regression modeling was performed to evaluated weight of independent predictors.

(B) Pre-treatment genetic alterations in > 5% of cases (n = 228). Bar plot showing the frequency of genetic alterations detected in Pre-treatment samples across 228 cases, color-coded by response category (CR, CRh, CRi, PR, NR, NA).

(C) A volcano plot summarizing univariate Fisher's exact test results for candidate variables. The x-axis represents the log2 odds ratio, and the y-axis is the −log10(*P*-value). Significant factors predicting lower CRc included *TP53*^{high}, *JAK2*, RAS-pathway genes, prior AZA exposure, and adverse karyotype. *DNMT3A*, *U2AF1*, and *ASXL1* demonstrated sub-

significant impact for favorable CRc. Among these analyses, eight factors satisfied the selection criteria ($P < 0.1$ or sufficient frequency) and were carried forward: −5/del(5q), 11p LOH, adverse karyotype, *ASXL1*, *DNMT3A*, prior AZA, RAS-pathway genes, and *TP53*^{high}. Detailed numerical results (odds ratio, 95% CI, *P*-values) are available in Supplementary Table 9.

(D) Parameter selection accounting for multiple collinearity. Bar plots showing gVIF values for candidate variables before (left) and after (right) adjustment. Variables with the second-highest gVIF were repeatedly removed until all remaining variables had gVIF ≤ 2. Of the eight univariate-selected factors, −5/del(5q) was excluded here due to collinearity. Consequently, seven variables (11p LOH, adverse karyotype, *ASXL1*, *DNMT3A*, prior AZA exposure, RAS-pathway genes, and *TP53*^{high}) proceeded to the next step.

(E) Regularization path for binomial Lasso regression. Lasso regression path plotted against the log of the regularization parameter (Log(λ)). Red points indicate cross-validated error rates, with the optimal λ value highlighted for variable selection in multivariable modeling. The seven variables from (D) were subjected to Lasso selection, and no additional factors were removed at this step.

(F) Multivariate logistic regression analysis for CRc. Forest plot summarizing the final logistic regression results for factors associated with complete data for response analysis (n = 180). Variables include 11p LOH, adverse karyotype, *ASXL1* mutations, *DNMT3A* mutations, prior AZA exposure, RAS pathway mutations, and *TP53*^{high}. The x-axis is log10 scaled.

VEN+AZA, venetoclax plus azacitidine; CR, complete remission; CRh, complete remission with partial hematological recovery; CRi, complete remission with incomplete hematological recovery; PR, partial remission; NR, no response; CRc, composite complete remission; SNV, single nucleotide variants; CNA, copy number alterations; SV, structural variants; RAS-pathway genes, mutations involving *NRAS, KRAS, PTPN11, CBL, NF1* genes; gVIF, generalized variance inflation factor; CI, confidence interval.

Supplementary Fig. 5: Supplementary information of survival analysis.

(A) PFS for patients in the cohort with available data (n = 227).

(B) RFS for the 136 cases that once achieved response (CRc or PR).

(C) OS of the entire cohort stratified by disease status at VEN+AZA administration. *P*-values are calculated with log-rank test.

PFS, Progression-free survival; RFS, relapse-free survival; CRc, composite complete remission; PR, partial remission; OS, overall survival; PIF, primary induction failure.

Supplementary Fig. 6: Analysis workflow and variable selection for identifying factors associated with OS in AML patients treated with VEN+AZA.

(A) A schematic flowchart illustrating the four-step analytic approach used to identify factors associated with OS in AML patients treated with VEN+AZA. In the first step, univariate Cox regression analyses were performed, and variables with $P < 0.1$ and sufficient frequency (≥10% for CNA, ≥5% for other alterations) were selected. In the second step, multiple collinearity was assessed using generalized variance inflation factors (gVIF ≤ 2). The third step involved Lasso regression-based parameter selection. Finally, a multivariate Cox regression model was constructed to identify independent predictors of OS.

(B) A volcano plot summarizing univariate Cox regression analysis results for candidate variables. The x-axis represents the log2 hazard ratio, and the y-axis is the −log10(*P*-value). Among these analyses, nine factors satisfied the selection criteria ($P < 0.1$ or sufficient frequency) and were carried forward: −5/del(5q), −7/del(7q), adverse karyotype, *BCORL1*, high age (≥ 70 years), MR-gene-alone, prior AZA exposure, RAS-pathway genes, and $TP53^{high}$. Detailed numerical results (hazard ratio, 95% CI, *P*-values) are available in Supplementary Table 10.

(C) Parameter selection accounting for multiple collinearity. Bar plots showing gVIF values for candidate variables before (left) and after (right) adjustment. Variables with the second-highest gVIF were repeatedly removed until all remaining variables had gVIF ≤ 2. Of the nine

univariate-selected factors, −5/del(5q) and adverse karyotype were excluded here due to collinearity. Consequently, seven variables (−7/del(7q), *BCORL1*, high age (≥ 70 years), MR-gene-alone, prior AZA exposure, RAS-pathway genes, and *TP53*^{high}) proceeded to the next step.

(D) Regularization path for Cox Lasso regression. Lasso regression path plotted against the log of the regularization parameter (Log(λ)). Red points indicate cross-validated error rates, with the optimal λ value highlighted for variable selection in multivariable modeling. The seven variables from (C) were subjected to Lasso selection, and −7/del(7q) was removed at this step. Consequently, six variables (*BCORL1*, high age (≥ 70 years), MR-gene-alone, prior AZA exposure, RAS-pathway genes, and *TP53*^{high}) proceeded to the next step, multivariate Cox regression model in Fig. 1D.

(E) Multivariate analysis of factors associated with OS. Forest plot summarizing multivariate Cox regression results for independent factors associated with complete data for survival analysis (n = 227). Key variables include *BCORL1* mutations, age (≥70 years), MR-gene-alone, prior azacitidine exposure, RAS-pathway genes, and *TP53*^{high}. The x-axis is log-scaled, and hazard ratios, CI, and *P*-values are provided for each variable.

VEN+AZA, venetoclax plus azacitidine; OS, overall survival; CR, complete remission; CRh, complete remission with partial hematological recovery; CRi, complete remission with incomplete hematological recovery; PR, partial remission; NR, no response; CRc, composite complete remission; SNV, single nucleotide variants; CNA, copy number alterations; SV, structural variants; RAS-pathway genes, mutations involving *NRAS, KRAS, PTPN11, CBL, NF1* genes; MR-gene, myelodysplasia-related gene; MR-gene-alone, patients classified as adverse-risk due to MR-gene mutations alone; gVIF, generalized variance inflation factor; CI, confidence interval.

Supplementary Fig. 7: Supplementary information of survival analysis.

(A) Kaplan–Meier curves showing PFS stratified by hematological response to VEN+AZA

treatment. *P*-values are calculated by log-rank test.

(B) A volcano plot showing the result of univariate analysis for OS. Achieving CRc has far more potent and significant impact on OS compared with other pre-treatment factors. The x-axis represents the log2 hazard ratio, and the y-axis is the −log10(*P*-value).

(C) Bar plots showing the change of mutation profile between Pre-treatment and Best-response phase. 89 cases were accounted for who achieved CRc and both Pre-treatment and Best-response phase samples were available.

(D, E) Kaplan–Meier curves showing OS (D) and RFS (E) stratified by molecular clearance of *TP53* mutations. *P*-values are calculated by log-rank test.

PFS, progression-free survival; OS, overall survival; RFS, relapse-free survival; CR, complete remission; CRh, complete remission with partial hematological recovery; CRi, complete remission with incomplete hematological recovery; PR, partial remission; SNV, single nucleotide variants; CNA, copy number alterations; SV, structural variants.

Supplementary Fig. 8: Oncoplots show the change in mutation profile between Pre-treatment and Best-response phases.

(A, B) These oncoplots show the change in mutation profile between Pre-treatment and Best-response phases. 80 cases treated with VEN+AZA (A) and 80 cases treated with intensive chemotherapy (B) who achieved CRc and have at least one mutated gene in either of Pre-treatment and Best-response phases are shown.

CR, complete remission; CRh, complete remission with partial hematological recovery; CRi, complete remission with incomplete hematological recovery.

Supplementary Fig. 9: Survival analysis stratified by residual mutations.

(A, B) Kaplan-Meier curves showing OS (A) and RFS (B) after VEN+AZA treatment and achieved CRc. The cases were stratified by the residual mutation status in the CRc phase: no residual mutation (blue) and DTA-mutations alone (green) in the CRc phase.

(C) Kaplan-Meier curves showing OS stratified by the residual mutations (red: residual non-DTA mutations, blue: no residual mutations or DTA-mutations alone) in CRc status. *P*-values are calculated by log-rank test.

OS, overall survival; RFS, relapse-free survival; DTA, *DNMT3A*, *TET2*, *ASXL1*.

Supplementary Fig. 10: Analysis of genetic alterations between Pre-treatment and Relapse phase samples.

(A) For each cell, the left upper triangles and the right lower triangles show the mutations in Pre-treatment and Relapse phase, respectively. The red and blue color of the rectangles shows that they constituted major or minor clones. Stacked bar graphs on the right show the number of patients having mutations in the corresponding genes.

(B) Box plots showing the number of genetic alterations per case (left: SNV, middle: CNA, right: SV). *P*-values are calculated with paired t-test. The 27 cases that relapsed after achieving CRc and had both Pre-treatment and Relapse samples analyzed.

SNV, single nucleotide variants; CNA, copy number alterations; SV, structural variants; CRc, composite complete remission.

Supplementary Fig. 11: Clonal shift from Pre-treatment to Relapse phase and survival outcomes in relapsed cases.

(A, B) Bar charts showing the mutation profile representing major clones for the cases that show clonal persistence **(A)** and clonal changes **(B)** in the Relapse phase samples.

(C, D) Kaplan-Meier curves showing OS (C) and RFS (D) stratified by the clonal shift patterns (red: clonal change, blue: clonal persistence). *P*-values are calculated by log-rank test.

OS, overall survival; RFS, relapse-free survival.

Supplementary Figure 1

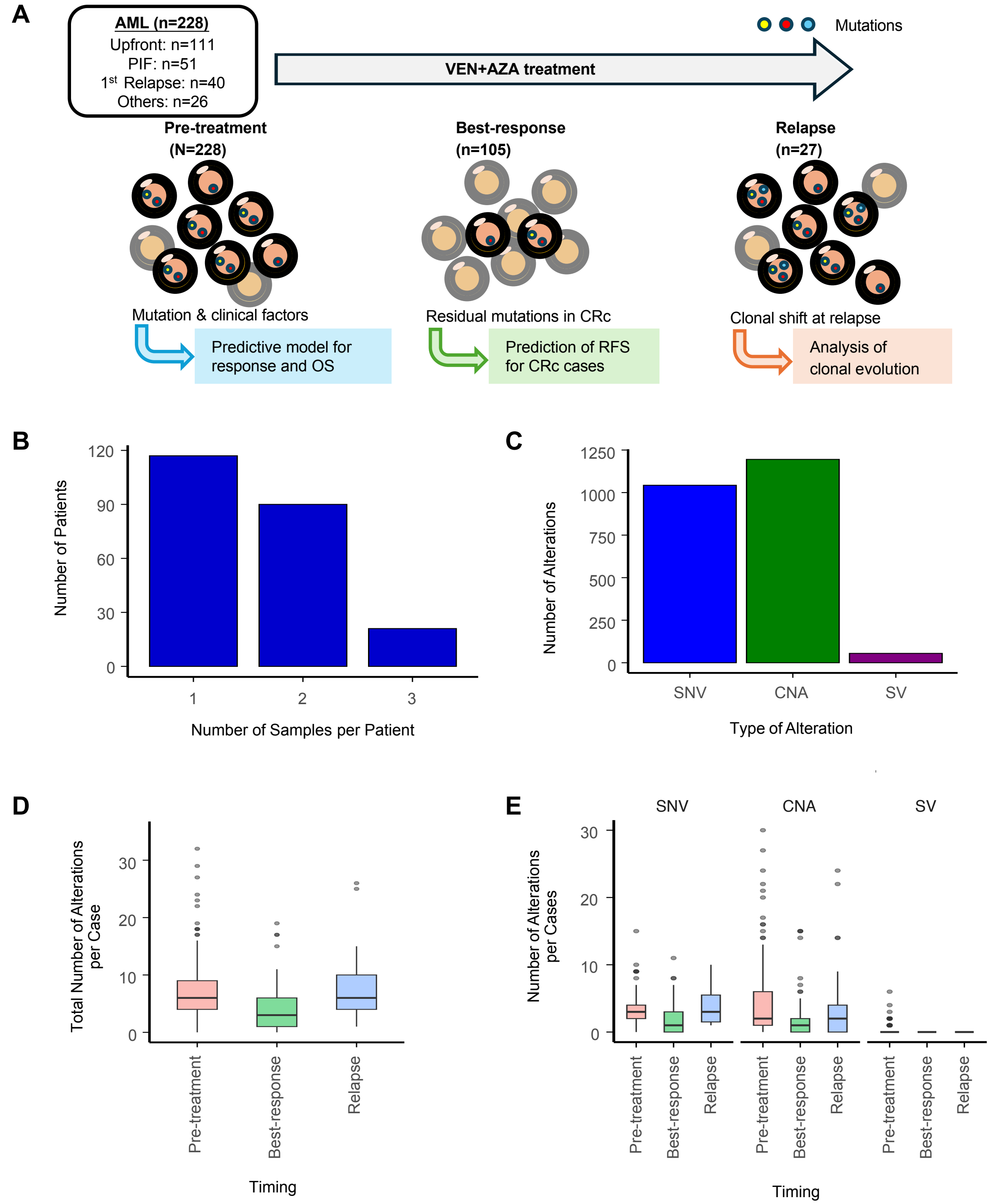

Supplementary Figure 2

A

		VAF Group	
		VAF ≥ 0.1	VAF < 0.1
**Allelic Group**	Multihit	41	0
	Monoallelic	6	4

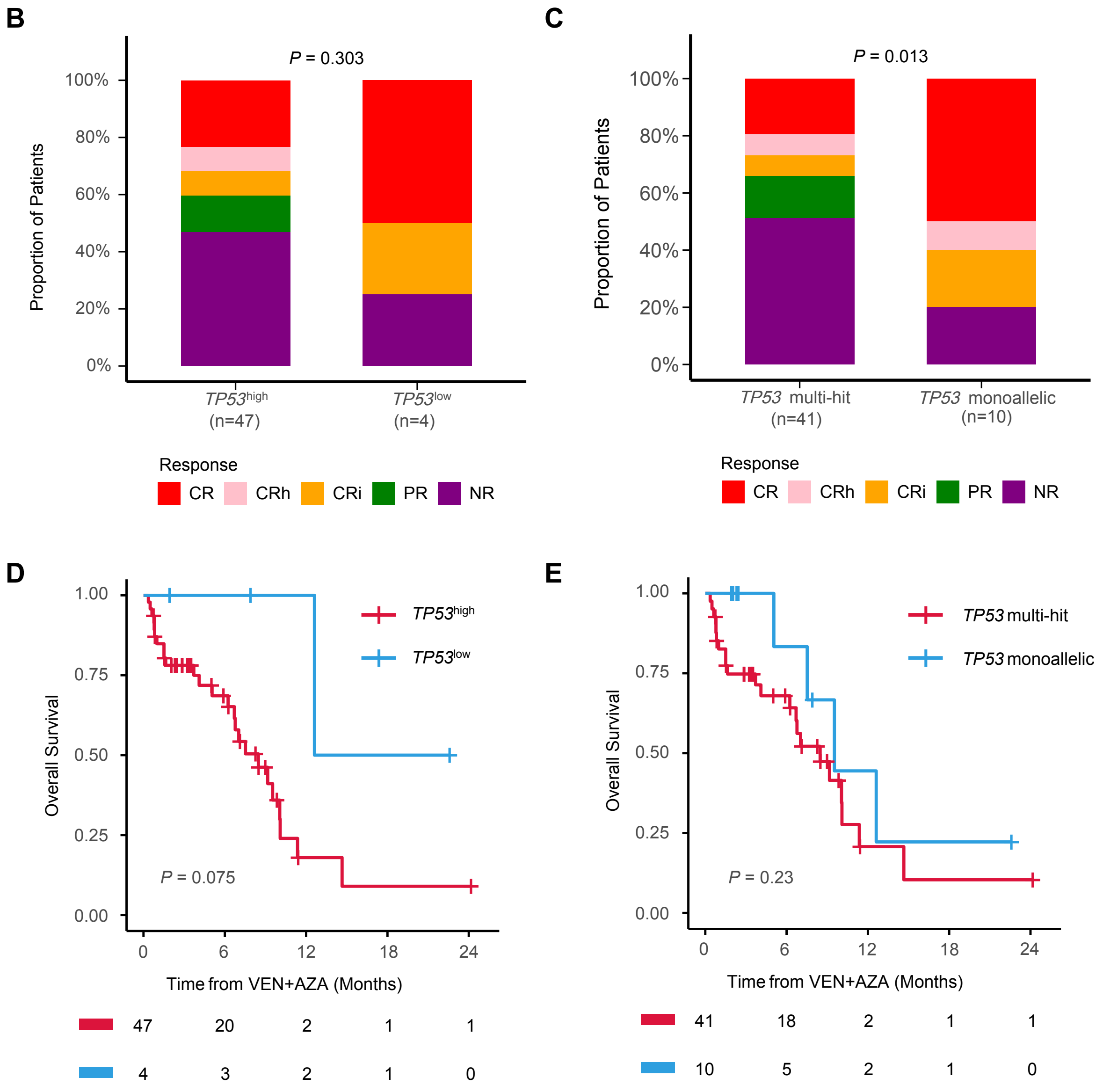

Supplementary Figure 3

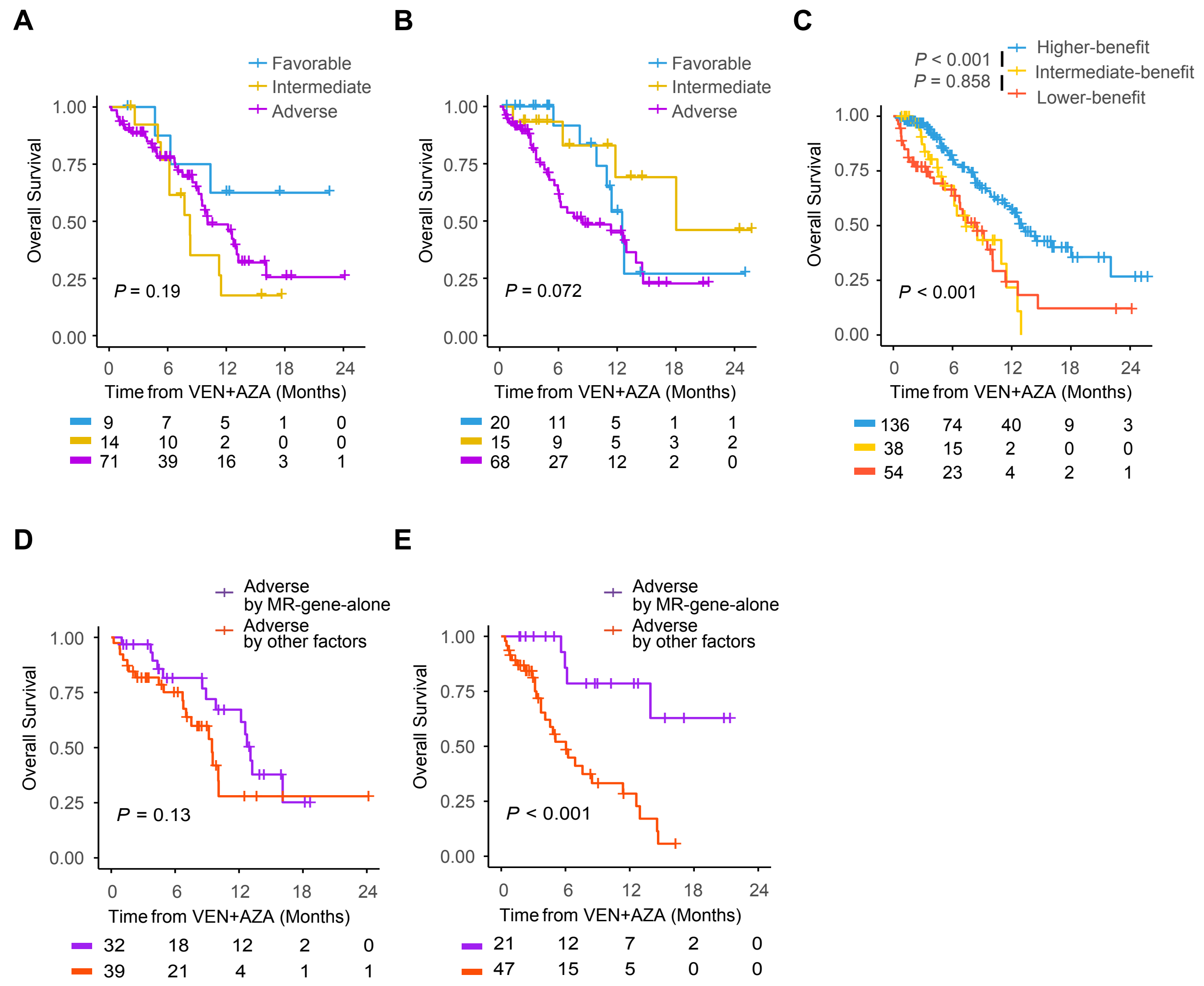

Supplementary Figure 4

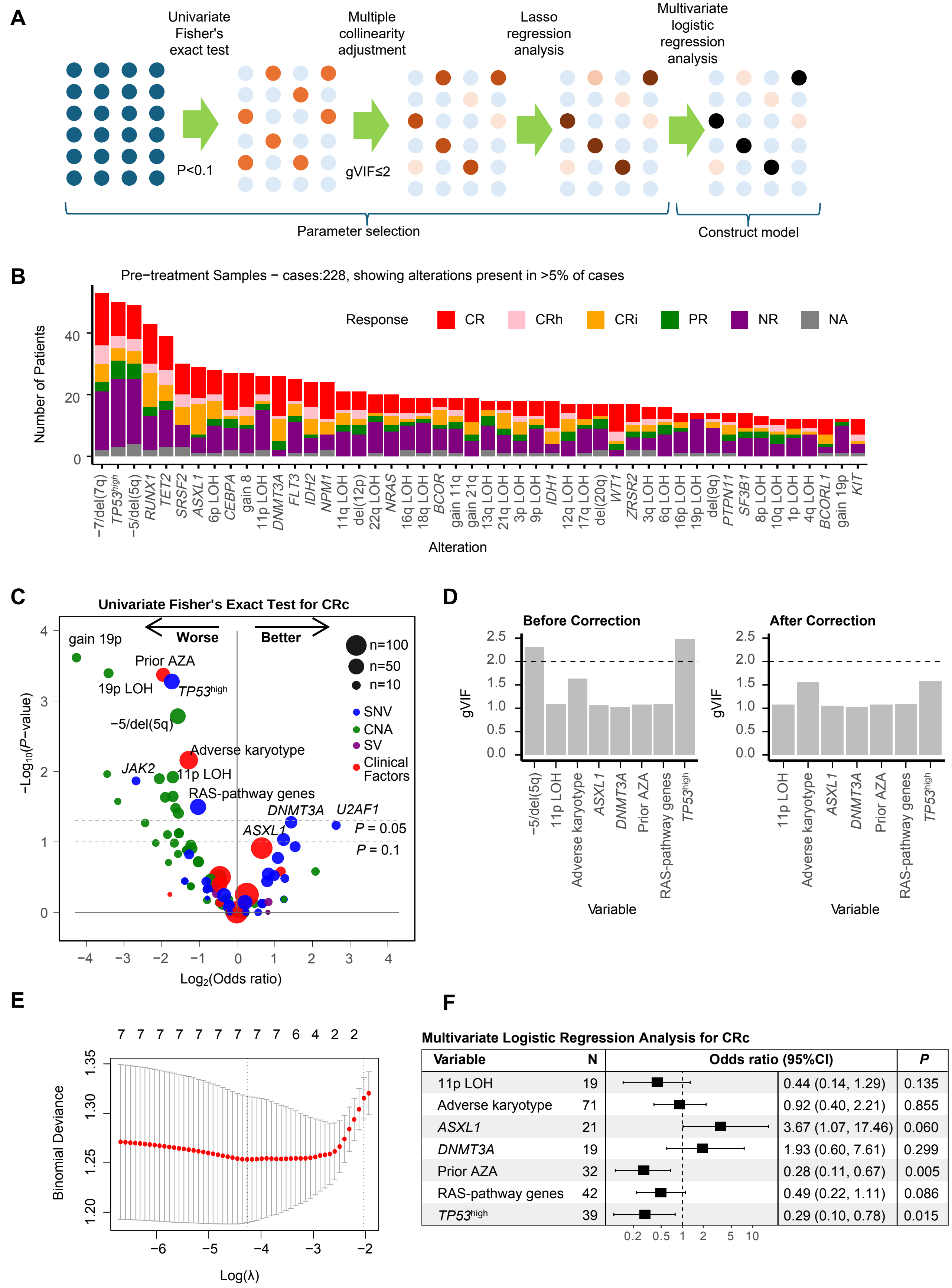

Variable	N	Odds ratio (95%CI)	P
11p LOH	19	0.44 (0.14, 1.29)	0.135
Adverse karyotype	71	0.92 (0.40, 2.21)	0.855
ASXL1	21	3.67 (1.07, 17.46)	0.060
DNMT3A	19	1.93 (0.60, 7.61)	0.299
Prior AZA	32	0.28 (0.11, 0.67)	0.005
RAS-pathway genes	42	0.49 (0.22, 1.11)	0.086
*TP53*high	39	0.29 (0.10, 0.78)	0.015

Supplementary Figure 5

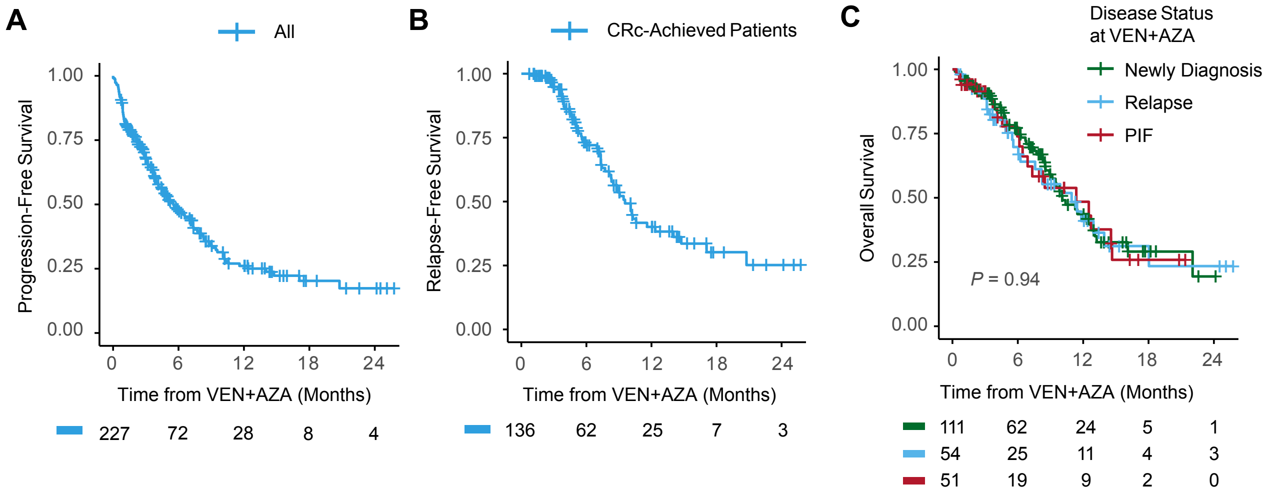

Supplementary Figure 6

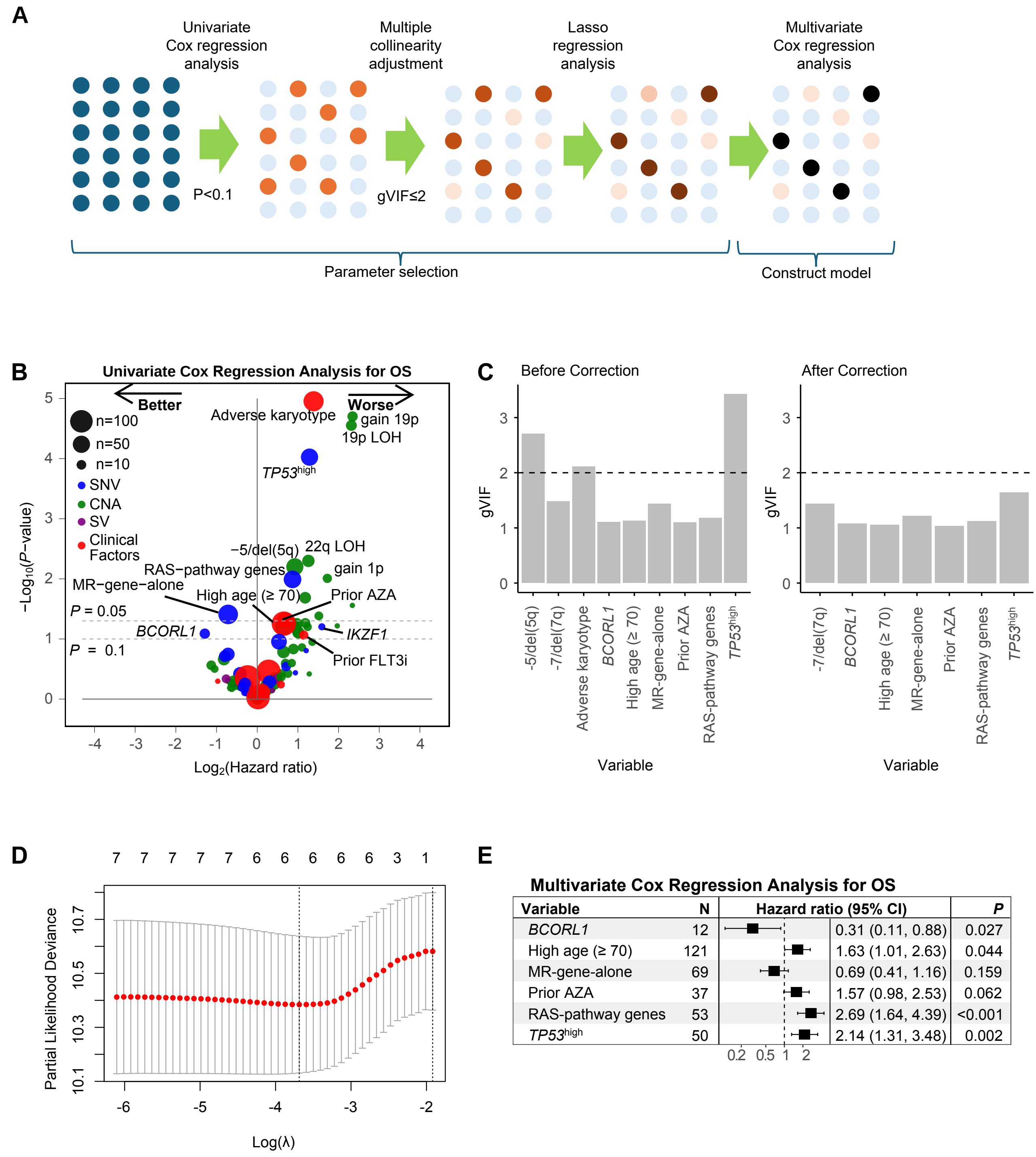

Supplementary Figure 7

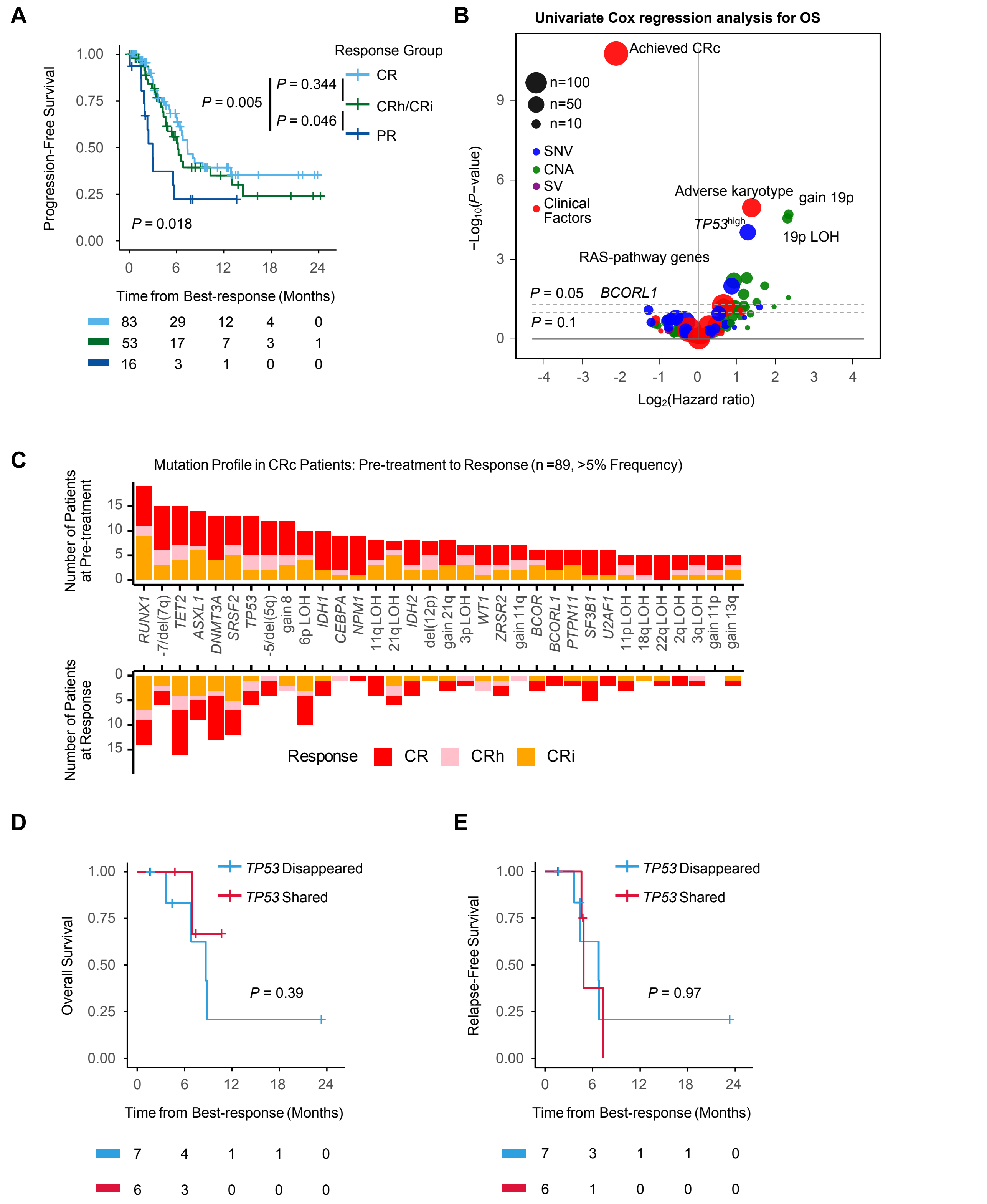

Supplementary Figure 8

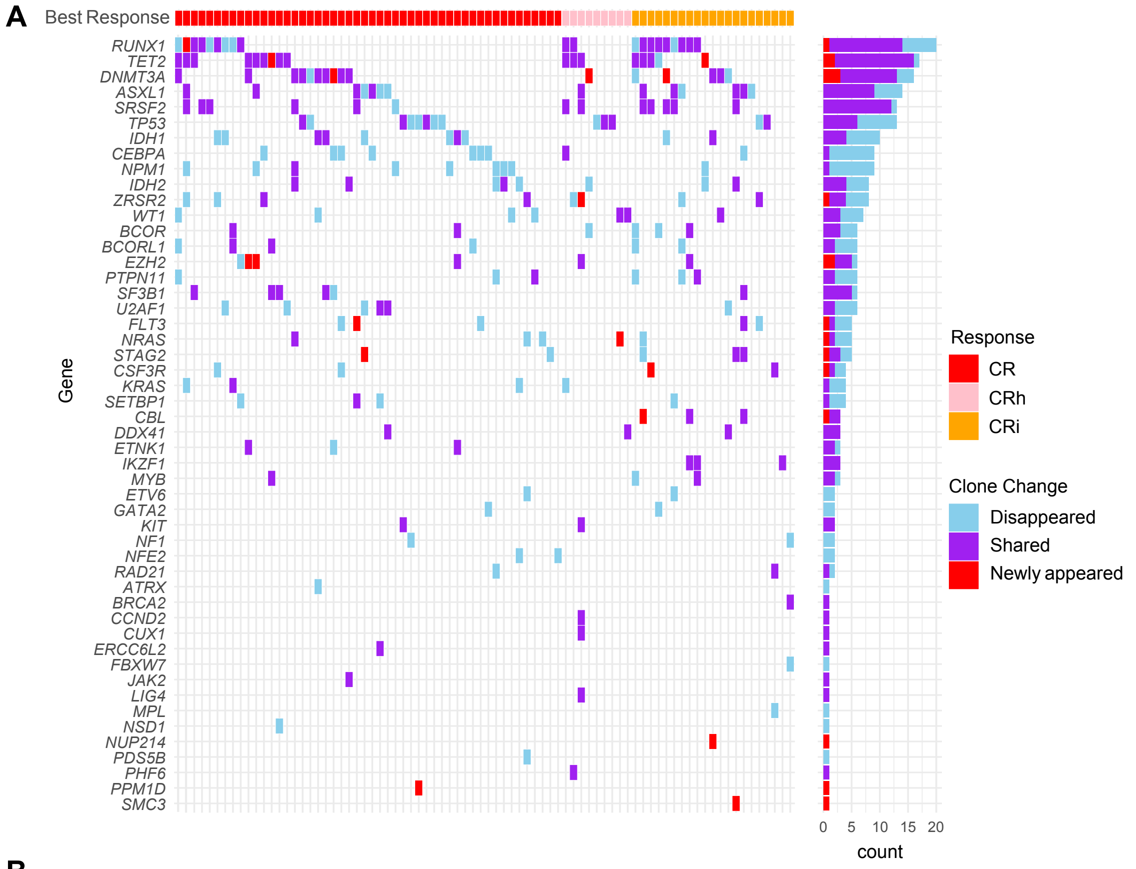

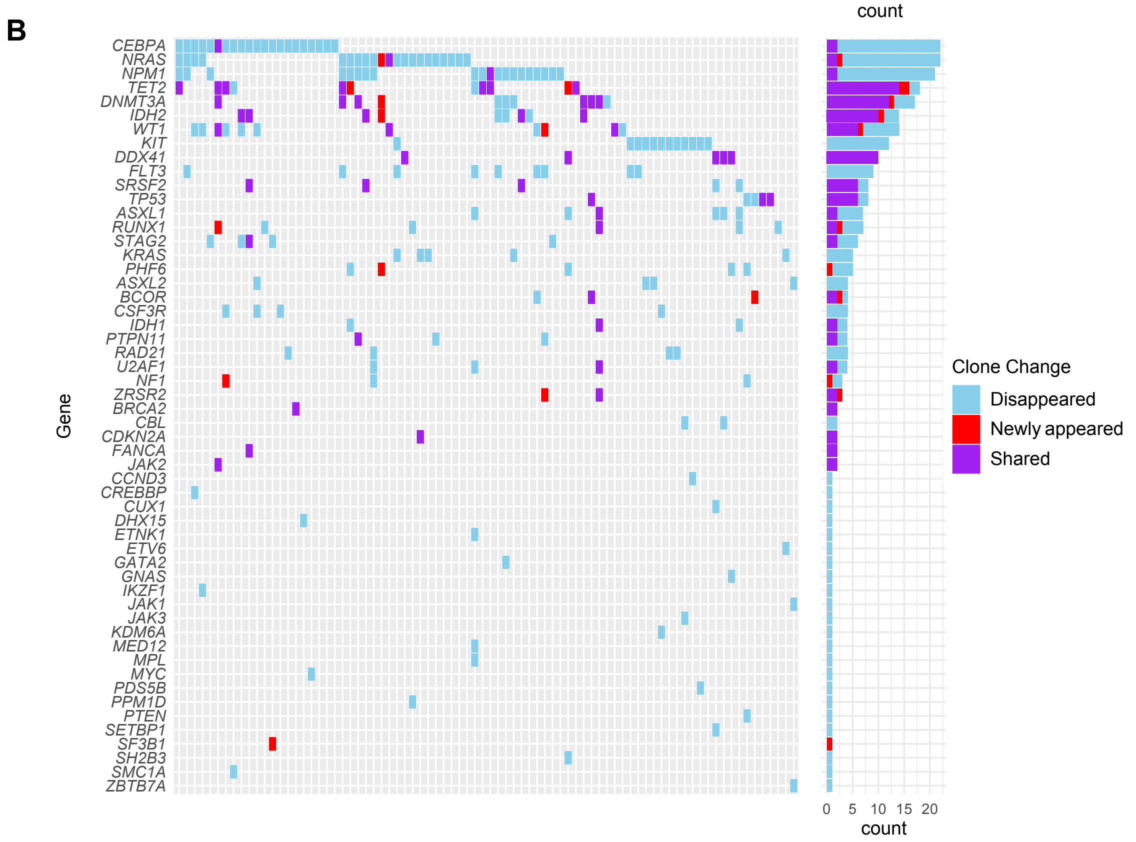

Supplementary Figure 9

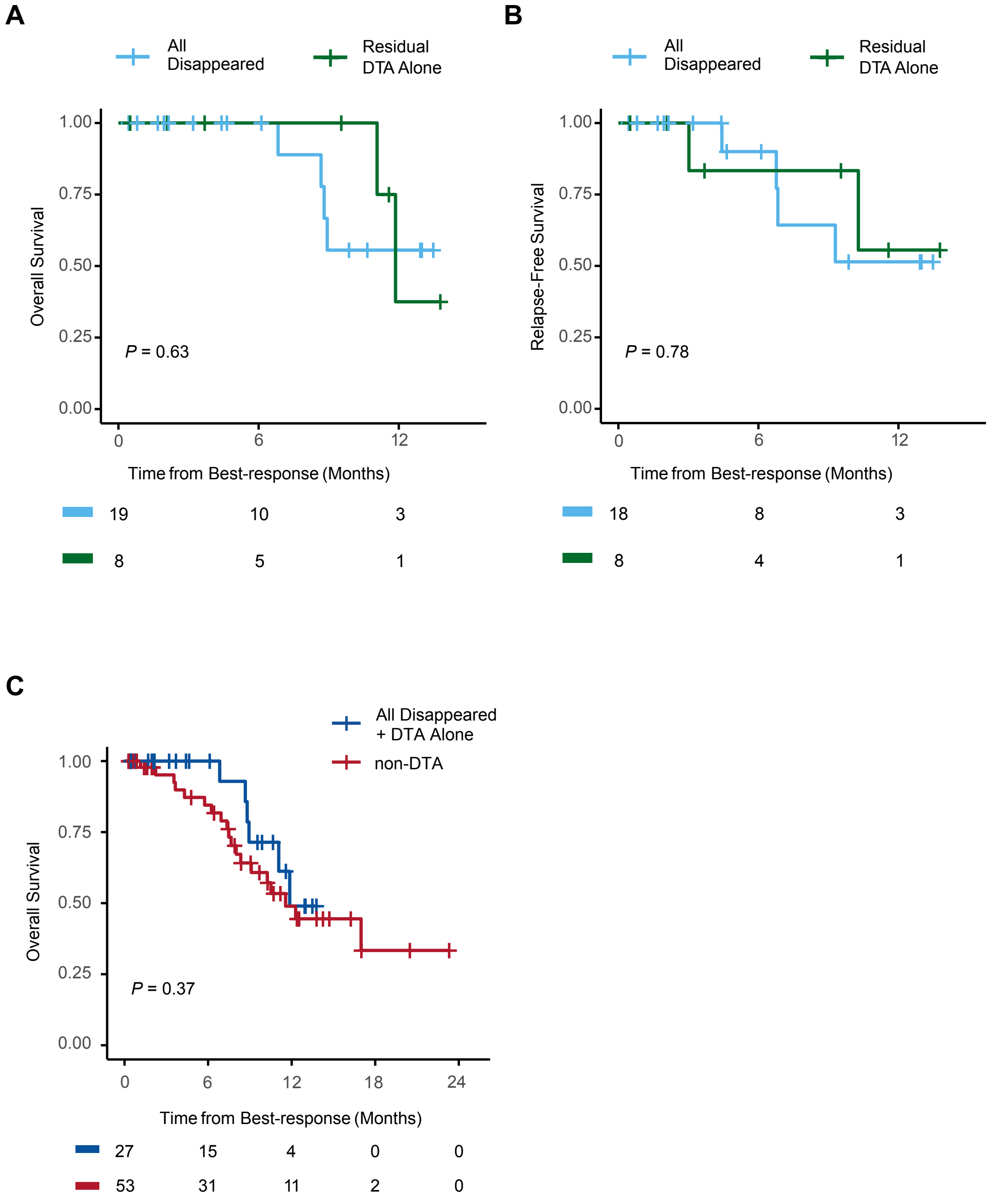

Supplementary Figure 10

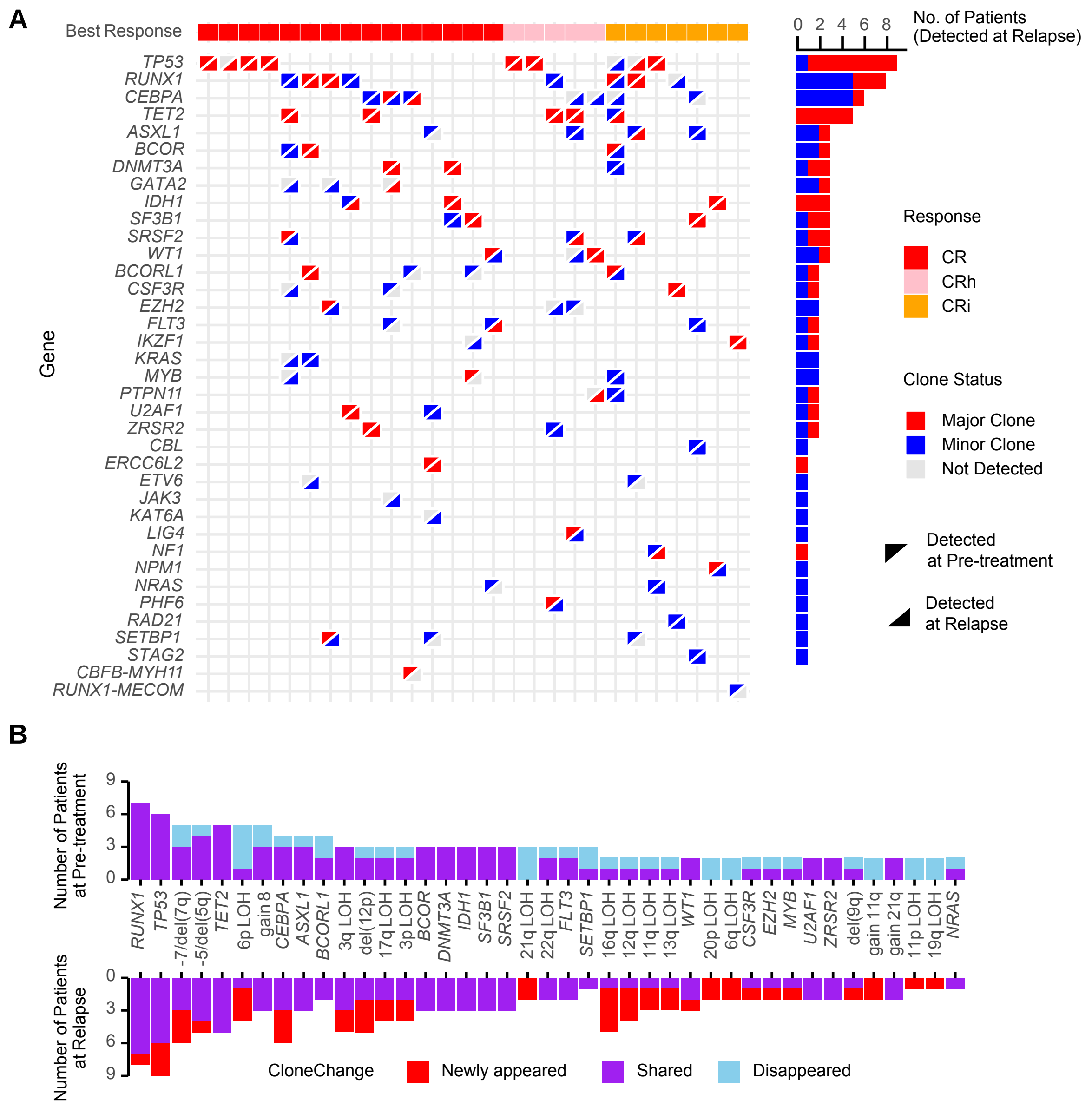

Supplementary Figure 11

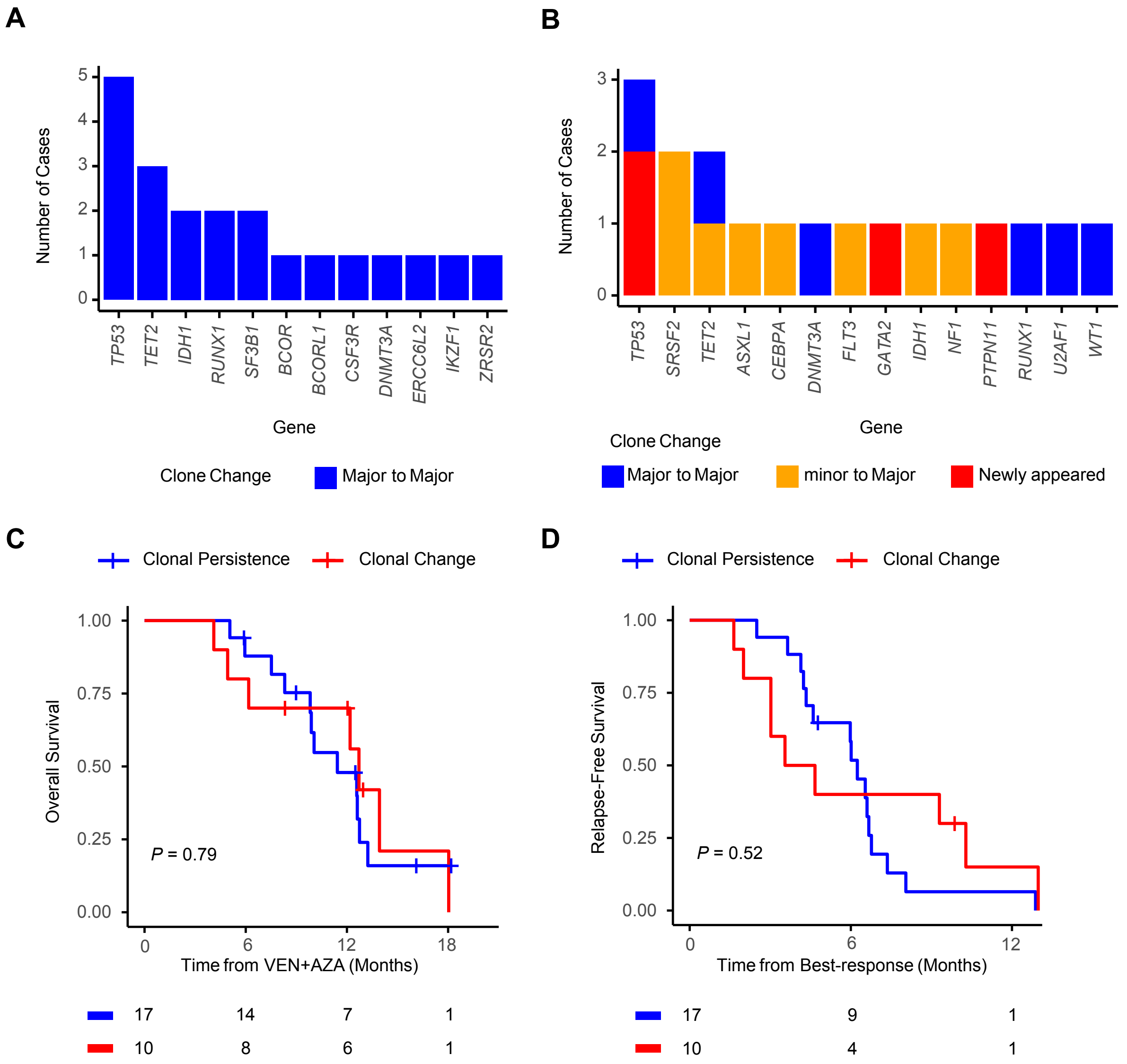

Supplement: Supplementary file 1 — Supplementary Information [file 41375_2025_2625_MOESM1_ESM.pdf]
